# Short- to mid-term clinical outcomes and survivorship of the Nexel total elbow arthroplasty in Japanese patients: a single-center retrospective study

**DOI:** 10.1016/j.jsea.2026.100006

**Published:** 2026-02-20

**Authors:** Yukihiro Kajita, Yohei Harada, Ryosuke Takahashi, Ryosuke Sagami, Yusuke Iwahori

**Affiliations:** a Department of Orthopaedic Surgery, Ichinomiya Nishi Hospital, Ichinomiya, Aichi, Japan; b Department of Orthopaedic Surgery, Hiroshima University, Hiroshima, Japan; c Department of Orthopaedic Surgery, Asahi Hospital, Gifu, Japan

**Keywords:** Total elbow arthroplasty, Japan, Nexel, Short- to mid-term outcomes, Survivorship, Complications

## Abstract

**Background:**

Total elbow arthroplasty (TEA) has become a well-established surgical option for advanced elbow disorders. The Nexel prosthesis (Zimmer Biomet, Warsaw, IN, USA) has been associated with mechanical complications and implant instability in previous international reports. However, no clinical outcomes have been published for Japanese patients. This study aims to evaluate the short- to mid-term clinical outcomes of the Nexel TEA in a Japanese cohort.

**Methods:**

We retrospectively reviewed 31 elbows in 30 patients (6 men, 24 women; mean age 73.0 years) treated with the Nexel prosthesis between 2015 and 2023, with a minimum follow-up of 24 months (mean 47.4 months). Primary diagnoses included rheumatoid arthritis (n = 9), fracture (n = 19), and osteoarthritis (n = 3) with some overlap. Clinical outcomes were assessed using the Mayo Elbow Performance Score, range of motion, and complication incidence.

**Results:**

The mean post-operative Mayo Elbow Performance Score was 79.3 points, reflecting generally favorable outcomes. Mean range of motion values were flexion 125.9°, extension −16.9°, pronation 76.5°, and supination 71.7°. Complications comprised aseptic loosening in 2 elbows, a periprosthetic olecranon fracture in 1, transient radial nerve palsy in 1, superficial infection in 3, and heterotopic ossification in 1. Most complications were managed successfully either conservatively or with minor revision.

**Conclusion:**

Despite prior concerns regarding early mechanical failure of the Nexel prosthesis, the present series demonstrates stable and satisfactory short- to mid-term outcomes in a Japanese patient population characterized by a high incidence of fracture indications and predominantly elderly, low-demand patients. These results may reflect careful surgical technique (including routine olecranon tip osteotomy to avert anterior flange impingement) and strict post-operative load-restriction protocols. Ongoing long-term follow-up and multicenter Japanese studies are required to confirm implant durability.

Total elbow arthroplasty (TEA) has become an established surgical intervention for patients with advanced elbow pathologies, including inflammatory arthritis, post-traumatic arthritis, and comminuted distal humeral fractures that cannot be reconstructed reliably with internal fixation.^15^, ^16^, ^17^ The principal objectives of TEA are the alleviation of pain, the restoration of an adequate arc of motion for activities of daily living, and the maintenance of joint stability. Over the past several decades, advances in implant design and the refinement of surgical techniques have led to improved outcomes and high levels of patient satisfaction.^12^^,^^28^ Nevertheless, compared with hip or knee arthroplasty, TEA continues to be associated with a higher complication profile, reflecting both the anatomical complexity of the elbow joint and the unique characteristics of the patient populations typically undergoing this procedure.^7^^,^^9^

Historically, many TEAs have been performed in patients with systemic inflammatory diseases, such as rheumatoid arthritis, who are often present with poor bone quality, ligamentous insufficiency, or multiple prior surgeries. Complications, including infection, implant loosening, instability, and periprosthetic fracture, were not uncommon in these patients.^22^ Among the available prosthetic options, the Coonrad-Morrey design, introduced in the early 1980s, represented a landmark innovation.^6^^,^^8^ Its semiconstrained "sloppy hinge" configuration allowed limited varus–valgus and rotational laxity, thereby distributing stress and providing a balance between stability and motion. As a result, the Coonrad-Morrey design has become one of the most widely adopted implants worldwide and has remained the gold standard for several decades. Despite its success, long-term follow-up revealed recurrent problems such as polyethylene wear, hinge-bushing failure, and mechanical loosening, underscoring the need for further improvement.^1^^,^^14^^,^^18^^,^^24^

In response to these limitations, the Nexel prosthesis (Zimmer Biomet, Warsaw, IN, USA) was introduced in 2014 as the successor to the Coonrad-Morrey implant. Several design modifications have been incorporated with the intention of enhancing durability and reducing wear. These included the use of vitamin E–treated, highly cross-linked polyethylene bushings to improve oxidation resistance, modifications of bushing geometry to reduce edge loading, and a posteriorly shifted axis of rotation intended to better approximate native elbow kinematics and facilitate extension. Importantly, these changes were implemented while retaining the semiconstrained philosophy of the original Coonrad-Morrey system.

However, despite these theoretical advantages, early clinical reports of the Nexel TEA have raised significant concerns. Several published studies from international cohorts have described unexpectedly high rates of mechanical failure, humeral component loosening, and the need for early revision.^19^^,^^25^ For example, revision and complication rates exceeding one-third of cases have been reported within only a few years of implantation, and overall prosthesis survival has been disappointingly low in comparison with its predecessor. Biomechanical analyses have suggested that a more posterior center of rotation may predispose patients to premature anterior impingement between the coronoid process or soft tissues and the humeral flange during flexion, resulting in repetitive pistoning, elevated stresses at the bone–cement interface, and eventual loosening.^13^

Despite these concerning reports, the Nexel prosthesis remains commercially available in multiple countries.^21^^,^^29^ To date, however, no outcome data have been published regarding its performance in Japanese patients. This lack of evidence raises challenges, as using published data from international cohorts may not always be applicable. Japanese patients generally present with smaller bone morphology and a higher prevalence of osteoporosis. Furthermore, TEA is often performed in Japanese patients for different indications, such as acute distal humerus fractures in elderly patients with low physical demand, rather than being performed as a primary TEA procedure for rheumatoid arthritis. These distinct demographic and clinical factors could influence both complication rates and implant survivorship.

Given this background, the present study was designed to evaluate the short- to mid-term clinical outcomes of the Nexel TEA in a Japanese population. Specifically, we aimed to investigate prosthesis survivorship, functional recovery, and complication rates, and to compare our findings with those of previously published international series. Through this analysis, we sought to clarify whether the complications reported internationally are also evident in Japanese patients or whether differences in patient characteristics and surgical practices may lead to a more favorable risk–benefit profile.

## Patients and methods

This study was a retrospective review of consecutive patients who underwent primary TEA using the Nexel prosthesis at our institution between 2015 and 2023. This research has been approved by the authors' affiliated institutions. The study was performed in accordance with the ethical standards outlined in the 1964 Declaration of Helsinki and its subsequent amendments. Written informed consent was obtained from all patients. All procedures were performed by an experienced upper extremity surgeon via a standardized surgical technique.

### Inclusion and exclusion criteria

Patients were eligible if they were aged 18 years or older and had a minimum clinical follow-up of 24 months. Patients were excluded if they had undergone revision TEA, conversion from hemiarthroplasty, or had incomplete clinical records that precluded adequate evaluation.

### Patient demographics

**Figure 1:**
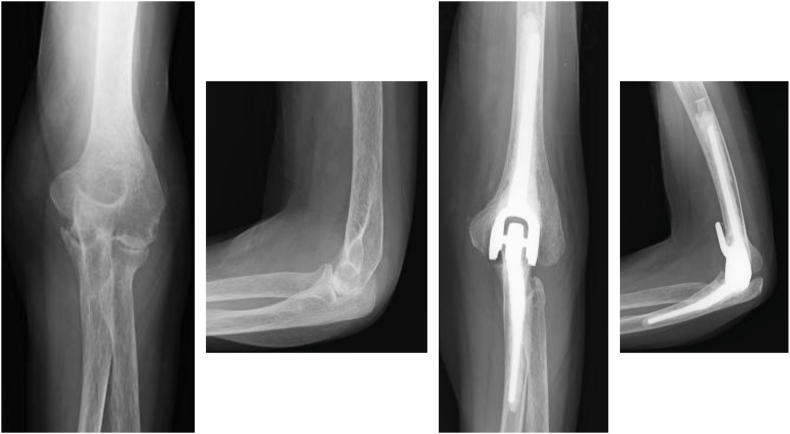
Total elbow arthroplasty was performed for rheumatoid arthritis.

**Figure 2:**
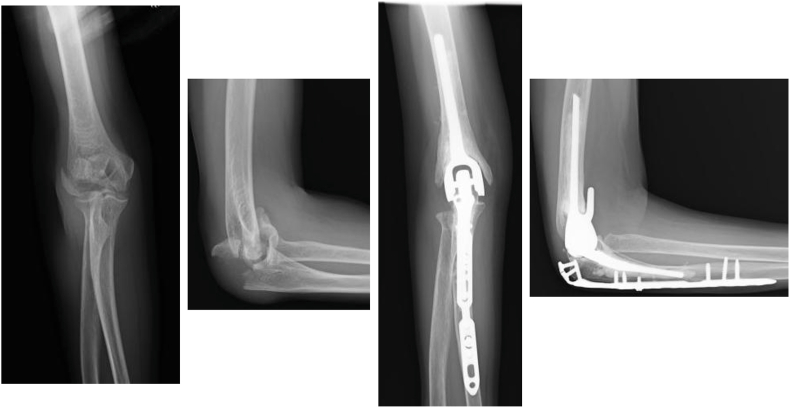
Total elbow arthroplasty was performed for a distal humeral fracture.

**Figure 3:**
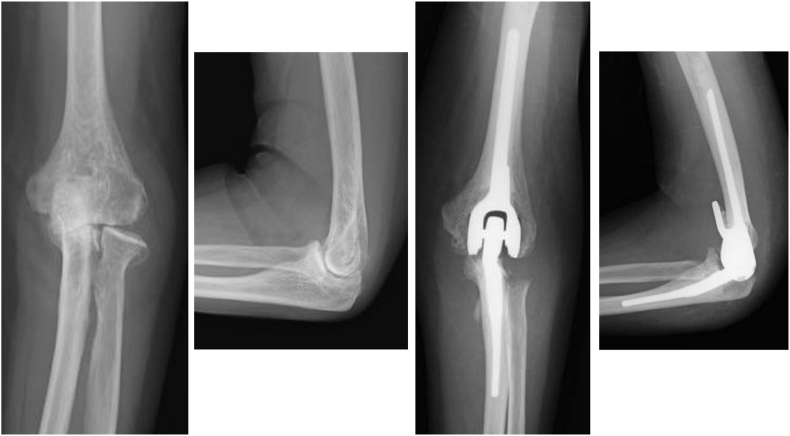
Total elbow arthroplasty was performed for osteoarthritis.

A total of 30 patients (31 elbows), consisting of 6 men and 24 women, with a mean age of 73.0 years (range, 47-91), met the inclusion criteria. The mean follow-up period was 44.4 months (range, 24-114). The primary diagnoses included rheumatoid arthritis (n = 9) (Fig. 1), fracture (n = 19) (Fig. 2), and osteoarthritis (n = 3) (Fig. 3), with some patients having overlapping etiologies.

### Clinical evaluation

Clinical outcomes were assessed via the Mayo Elbow Performance Score (MEPS) and active range of motion (ROM) (flexion, extension, pronation, and supination) measured at final follow-up. Active ROM was measured in all patients by physical therapists using a universal goniometer. Elbow flexion and extension were assessed with the patient in the supine position, while forearm pronation and supination were measured with the patient in the seated position with the elbow flexed at 90°. Complications, including aseptic loosening, periprosthetic fracture, nerve injury, infection, and heterotopic ossification, were recorded from medical records.

### Radiographic evaluation

Standard anteroposterior and lateral radiographs of the elbow were obtained immediately after surgery and at the most recent follow-up. Radiographs were reviewed for evidence of aseptic loosening or periprosthetic lucency using previously established criteria.^10^ Loosening was defined as gross migration or change in implant position, or the presence of continuous radiolucent lines >1 mm at the implant–cement or bone–cement interface surrounding the component.^26^

### Statistical analysis

Descriptive statistics were used to summarize demographic data, clinical outcomes, and complication rates. Continuous variables were expressed as mean ± standard deviation, and categorical variables as counts and percentages.

### Surgical technique

All procedures were conducted under general anesthesia with the patient placed in the lateral decubitus position. A single senior surgeon with subspecialty training in elbow surgery performed all operations. A posterior skin incision and a triceps-sparing Campbell approach were used in every case. The ulnar nerve was identified and routinely transposed anteriorly.

**Figure 4:**
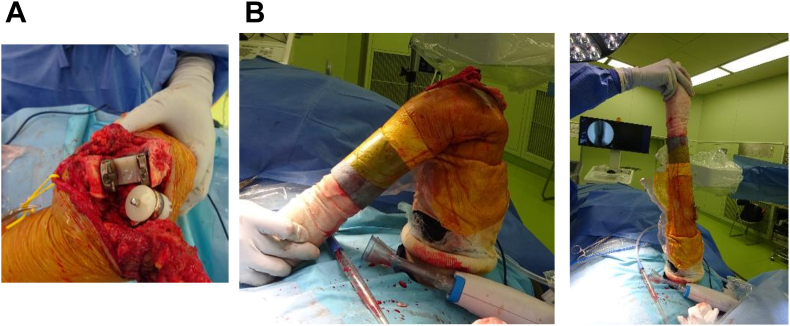
Surgical technique. (**A**) Both humeral and ulnar components were fixed with bone cement. (**B**) The humeral and ulnar components were docked, and intraoperative fluoroscopy confirmed that flexion and extension were achievable. When maximum flexion caused impingement between the anterior flange and the coronoid process, additional resection of the coronoid process was performed.

A Nexel TEA was implanted in all patients. Bone preparation and component positioning were carried out in accordance with the manufacturer's guidelines. Both the humeral and ulnar components were fixed via antibiotic-loaded, low-viscosity cement applied with a pressurized system designed for narrow medullary canals, ensuring an adequate cement mantle (Fig.4, *A*). When maximum flexion resulted in impingement between the anterior flange and the coronoid process, approximately 5 mm of the coronoid was resected to prevent anterior pistoning (Fig.4, *B*). A closed-suction drain was placed and maintained until splint removal to reduce hematoma and swelling. The triceps mechanism was preserved through the Campbell approach, and layered closure was performed without detachment of the extensor mechanism. No autologous bone grafting was required in this series.

Post-operatively, all elbows were immobilized in extension with a posterior splint for 2 days. All patients followed a standardized rehabilitation protocol under the guidance of a physical therapist. Gentle active-assisted ROM exercises were initiated on post-operative day 3, including elbow flexion–extension and forearm pronation–supination. During the first 6 weeks, progressive ROM exercises were performed without resistance. At 6 weeks post-operatively, mild resistance exercises were introduced for activities of daily living, with gradual progression based on individual patient tolerance and healing status. To minimize mechanical stress on the implant, received lifelong activity restrictions were prescribed: no lifting exceeding 5 kg for single efforts and no repetitive lifting exceeding 1 kg. High-demand activities, including pushing, pulling, upper extremity weight-bearing, and impact loading, were permanently restricted.

## Results

The mean post-operative MEPS was 79.3, indicating overall good function. The mean active ROM was as follows: 125.9° of flexion, −16.9° of extension, 76.5° of pronation, and 71.7° of supination.

Complications were relatively uncommon in this series (Table I). Aseptic humeral component loosening occurred in 2 elbows (6.5%), whereas one patient sustained a post-operative olecranon fracture that healed successfully with conservative management. Transient radial nerve palsy was noted in one patient, and heterotopic ossification developed in another. Superficial infections were observed in 3 elbows, all of which were successfully managed with surgical debridement. No cases of deep infection or catastrophic implant failure occurred during the follow-up period. Overall, implant survivorship was favorable, with all prostheses remaining in situ, and no revisions for aseptic loosening were needed.

**Table I:** Summary of complications.

Complication	n (%)
Aseptic humeral component loosening	2 (6.5)
Periprosthetic olecranon fracture	1 (3.2)
Transient radial nerve palsy	1 (3.2)
Superficial infection	3 (9.7)
Heterotopic ossification	1 (3.2)
**Total**	**8 (25.8)**

## Discussion

This study provides the first comprehensive report on the clinical performance of the Nexel TEA in a Japanese patient population. Whereas prior international publications have generated substantial concern regarding the survivorship of this implant,^19^^,^^25^ our results demonstrated overall favorable short- to mid-term outcomes, with high functional recovery, a low incidence of revision, and an acceptable complication profile. These findings are particularly noteworthy given that several well-known published data from international cohorts have documented alarmingly high rates of mechanical failure, loosening, and early revision associated with the Nexel prosthesis.^19^^,^^25^

The controversy surrounding the Nexel implant largely originates from a report by Morrey et al,^19^ who reported a revision rate exceeding 30% within only a few years, primarily due to humeral-component loosening. Their analysis suggested multiple mechanisms, including osteolysis related to the titanium-on-polyethylene articulation, accelerated polyethylene wear, and, most importantly, anterior impingement between the humeral flange and the coronoid process. This phenomenon was theorized to create a pistoning effect, whereby abnormal axial and rotational stresses were transmitted to the cement–bone interface, leading to loosening of the humeral or ulnar components.^4^^,^^11^ The posteriorly positioned center of rotation of the Nexel prosthesis, although intended to facilitate elbow extension, has been implicated in exacerbating this impingement by promoting premature contact between the ulna and the humeral flange during flexion.^13^ Subsequent fatigue failure at the cement–bone interface or at the porous-coated Ti-6Al-4V surface has been described as a critical factor driving early mechanical breakdown.

In contrast, Owen et al^21^ reported favorable outcomes in 11 Nexel TEAs with a mean follow-up of 53.3 months, with 100% implant survivorship and no revisions or reoperations. Complications included one case of radiographic loosening limited to the humeral component (9.1%) and one transient ulnar nerve neuropraxia without permanent dysfunction (9.1%). The mean MEPS was 90 points, with 73% of patients reporting satisfactory outcomes. Importantly, their radiographic evaluations did not reveal significant component loosening or bushing wear. However, the small sample size of this study limited the generalizability of its conclusions, underscoring the heterogeneity of reported outcomes and the likelihood that surgical technique, patient selection, and post-operative management play crucial roles in determining implant survivorship.

To mitigate the small sample size and relatively short follow-up period of previous reports, a recent review of the New Zealand National Joint Registry evaluated long-term survivorship and revision rates for both Nexel and Coonrad-Morrey TEAs.^29^ This large, prospectively collected dataset demonstrated that, over a 23-year period, the Nexel prosthesis had comparable implant survival and revision rates to those of the well-established Coonrad-Morrey system. The reported 5-year revision rates were 7.3% for Nexel and 4.5% for Coonrad-Morrey, with an average time to revision of 3.1 and 4.9 years, respectively.^19^^,^^25^ These findings suggest that, in a real-world national cohort, the Nexel TEA can achieve reliable mid-term outcomes similar to those of its predecessor, despite earlier reports indicating high failure rates. The authors highlighted that differences in surgical technique and post-operative management are likely to contribute to the observed variability, emphasizing the importance of careful implantation and follow-up.

This study contributes to the international discourse on the Nexel TEA by providing additional evidence from a Japanese cohort with a high proportion of distal humerus fracture cases. Unlike many published data from international cohorts^5^^,^^22^ where inflammatory arthritis has historically been the most common indication for TEA, the majority of our procedures were performed for complex fracture scenarios. Despite the well-known technical difficulties associated with fracture-related cases,^23^^,^^27^ such as bone loss, instability, and higher risk of component malposition, our outcomes were generally favorable. This finding suggests that, with careful and precise surgical technique, the Nexel implant remains a reliable option even in these challenging clinical settings.

**Figure 5:**
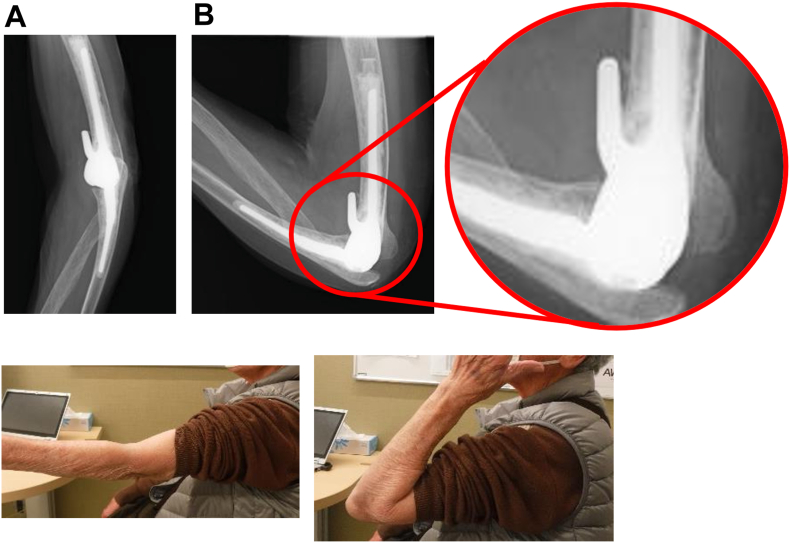
(**A**) Plain radiograph of the elbow in maximum extension. (**B**) Plain radiograph of the elbow in maximum flexion showing no impingement between the anterior flange and the coronoid process.

A key element of our technique that may have influenced the results was the routine intraoperative assessment of anterior impingement (Fig. 4). When maximum elbow flexion revealed contact between the humeral flange and the coronoid process, we performed a limited resection of approximately 5 mm of the olecranon tip. This maneuver was designed to create accommodation for the coronoid during deep flexion, thereby mitigating the pistoning mechanism described in previous literature (Fig. 5).^4^^,^^11^ Such coronoid accommodation is a feature incorporated into other implant designs, such as the Latitude and Discovery prostheses, which may explain their lower reported failure rates despite also employing a posterior center of rotation.^13^^,^^20^ We believe that this technical modification was a critical factor in reducing abnormal stresses on the cement–bone interface and contributed to the low incidence of humeral loosening in our cohort.

Other aspects of perioperative management also likely played a role in our positive outcomes. All surgeries, including routine anterior transposition of the ulnar nerve and careful cementing under pressurization, were performed by a single, high-volume elbow surgeon via a standardized technique. Uniform post-operative rehabilitation protocols were implemented, ensuring compliance with lifting restrictions to limit excessive mechanical loading during the vulnerable early healing phase. Furthermore, our patient population consisted primarily of elderly, low-demand individuals, who typically generate less stress across the implant than younger or more active patients who are frequently included in published data from international cohorts. Implant survivorship remained consistent in higher-risk subgroups, including individuals with post-traumatic arthritis, rheumatoid arthritis, and obesity at a young age. This suggests that the surgical procedure and post-operative care may have a greater impact than these adverse baseline factors. Nevertheless, some caution is warranted in interpreting these results. First, our analysis was retrospective in nature and involved a relatively small sample size, limiting the statistical power. Second, unlike some published data from international cohorts,^2^^,^^3^ we did not incorporate systematic radiographic follow-up of asymptomatic patients. As such, we may have underestimated the prevalence of subclinical component loosening. Third, the discrepancy between our favorable outcomes and the poor survivorship reported by others may partly reflect these methodological differences, as well as variations in surgeon experience. It has been suggested that high-volume, expert surgeons may be more adept at detecting subtle radiographic signs of early loosening and therefore intervene earlier with revision surgery, potentially inflating revision rates in those series. Fourth, our findings are specific to the Nexel total elbow prosthesis and may not be generalizable to other implant designs due to differences in design features such as center of rotation, bushing geometry, and polyethylene formulation.

Taken together, our findings indicate that the Nexel TEA, although criticized for high complication and revision rates internationally,^19^^,^^25^ can achieve satisfactory short- to mid-term outcomes when careful surgical technique and strict post-operative protocols are applied. The deliberate effort to reduce anterior impingement, along with consistent cementing methods and cautious patient management, appears to be central to achieving implant stability. Moving forward, multicenter collaborative studies with longer-term follow-up and standardized radiographic monitoring will be essential to determine whether these favorable results persist over time and to clarify which technical variables most strongly influence implant survivorship.

## Conclusion

The Nexel TEA demonstrated favorable short- to mid-term outcomes in a Japanese patient population that included a high proportion of fracture cases and predominantly elderly, low-demand patients. Careful surgical technique, including olecranon tip osteotomy to prevent anterior flange impingement and adherence to post-operative load restrictions, contributed to stable implant performance and low complication rates. Long-term follow-up is needed to confirm these findings and assess implant survivorship.

## Disclaimers:

Funding: No funding was disclosed by the authors.

Conflicts of interest: The authors, their immediate families, and any research foundations with which they are affiliated have not received any financial payments or other benefits from any commercial entity related to the subject of this article.
